# Asthma: From Phenotypes to Personalized Medicine

**DOI:** 10.3390/jpm12111853

**Published:** 2022-11-06

**Authors:** Petros Bakakos

**Affiliations:** 1st University Department of Respiratory Medicine, National and Kapodistrian University of Athens, 11527 Athens, Greece; petros44@hotmail.com

Asthma is a heterogeneous disease of the airways with a high prevalence worldwide characterized by chronic inflammation. The aim of asthma management is the control of the disease, and the cornerstone of asthma treatment is inhaled corticosteroids [1].

Asthma is no longer recognized as a unique manifestation, and the “one size fits all” approach may apply only in the treatment of mild asthma.

The use of as-needed treatment containing inhaled corticosteroids (ICS) plus fast-acting bronchodilators (either short or the long-acting formoterol) in mild asthma is the dominant change in asthma management, and both randomized and real-world studies favor such an approach and associate it with fewer exacerbations and good asthma control [2]. The fact that asthma control was slightly better with daily inhaled corticosteroids compared to as needed ICS-formoterol is compensated by the low adherence that most mild asthmatics might demonstrate due to the cessation of treatment in view of minor symptoms [3,4].

As asthma becomes less well-controlled and more severe (moderate-to-severe) medications are becoming less effective. Phenotypes and endotypes are known as pathologic and molecular features, respectively, that might not be directly associated with each other but may define a better response to treatment, and that is important from a clinical point of view. Accordingly, it has become necessary to define the phenotype of a severe asthmatic patient mainly based upon biomarkers and clinical features, and that has consequently led to treatable traits and personalized medicine. Each asthmatic, and especially the severe one, is evaluated in terms of phenotype followed by the initiation of a treatment regimen that would be more effective in improving symptoms and/or reducing exacerbations, both of which are considered measures of control [5].

At the same time, the effort to reduce the use of oral corticosteroids as maintenance treatment in severe asthma was dictated by their serious side effects and was accomplished to a significant degree with the initiation of biologics [2]. At the moment there are six monoclonal antibodies for the treatment of severe asthma: omalizumab, mepolizumab, reslizumab, benralizumab, dupilumab, and tezepelumab.

Allergic asthma remains the most common asthma phenotype and is characterized by type 2 airway inflammation and IgE sensitization to allergens. It usually arises in childhood, and asthmatic symptoms typically present after exposure. Biologics have been added in the treatment armamentarium for allergic asthma along with allergen avoidance measurements and allergen-specific immunotherapy [6]. An important issue regarding biologics for allergic asthma is the possible disease-modifying effect mounting to the question for the duration of such treatment. Allergen immunotherapy is the only etiological therapy and has been suggested to modify the course of allergic disease in children by preventing the progression from allergic rhinitis to allergic asthma [7].

In severe eosinophilic asthma, eosinophils are the hallmark of this prevalent phenotype in which other cells such as T cells (T helper 2 (Th2) and type 2 innate lymphoid cells are involved. For severe eosinophilic asthma, targeted therapies directed against IL-5/IL-5R and IL-4R have proved effective in the reduction in asthma exacerbations but also in the improvement of asthma control and lung function [8]. Unanswered questions such as the duration of treatment, the possible combination of biologics in overlapping phenotypes, and, again, the disease-modifying effect remain. In case of an inadequate response to a biologic, there is now the possibility to switch to another, and real-life data of such switches have been published [9]. Still, the initial choice of the most proper biologic remains a clinical challenge preceded by the possibly better phenotyping of the patient.

T2-low asthma remains a challenging phenotype with limited therapeutic options. The anti-TSLP mAb tezepelumab has shown some efficacy in T2-low asthma and has been approved for severe asthma with no particular phenotype limitation [9]. The so-called alarmins TSLP, IL-33, and IL-25 are produced by bronchial epithelial cells and novel antibodies directed against alarmins are under investigation for severe asthma including T2-low asthma.

Chronic rhinosinusitis with nasal polyps (CRSwNP) is a chronic inflammatory disease and it is common in patients with asthma and, particularly, severe asthma. Severe asthma is effectively treated with biologics and the coexistence of severe asthma with CRSwNP presents a phenotype that is more likely to respond to such treatment, showing a reduction in asthma exacerbation rate, reduced maintenance corticosteroids, and improvement in asthma control, lung function, and asthma-related quality of life [10]. Recently, patients with CRSwNP and no asthma or comorbid mild-to-moderate asthma also became eligible for biologic treatments. While in the case of severe asthma with CRSwNP, the asthma phenotype could guide the initial biologic choice, there is neither clear evidence nor biomarkers for such choice in CRSwNP with or without asthma. Moreover, there are no switching studies from one biologic to another at the moment.

The classification of four inflammatory asthma phenotypes based upon induced sputum—the neutrophilic, the eosinophilic, the mixed, and the paucigranulocytic—has aided personalized treatment through the recognition of the dominant inflammatory pattern and its underlying mechanisms [11]. The most intriguing of these four subtypes is the paucigranulocytic. The paucigranulocytic asthma phenotype remains a mystery in terms of underlying mechanisms and pathobiology. Despite being described in some cases as a previous eosinophilic phenotype with a good response to anti-inflammatory treatment, it seems that other mechanisms and inflammatory cells are also involved. The problem is that many patients with paucigranulocytic asthma remain uncontrolled and experience exacerbations, irrespective of the low grade of airway inflammation [12]. While the true mechanisms that drive the dissociation of airway inflammation with airway obstruction and hyperresponsiveness are not clear, the more or less expected lack of corticosteroid efficacy imposes the need for other pharmacologic but also non-pharmacologic treatments such as smoking cessation, bronchial thermoplasty, and weight reduction. The recognition of asthmatics with paucigranulocytic asthma is important as it may aid the reduction in useless high doses of inhaled corticosteroids despite the difficulty in not prescribing ICS in a severe asthmatic from the clinician’s perspective.

Currently, approximately one-fourth of asthma patients are smokers. The smoking asthmatic presents a phenotype characterized by accelerated lung function decline, non-reversible airflow limitation, increased symptoms, more exacerbations, and impaired quality of life [13]. Sometimes, the distinction of the smoking asthmatic from the chronic obstructive pulmonary disease (COPD) patient is challenging, especially in view of the possibility of asthma with fixed airflow obstruction and COPD with significant bronchodilator reversibility. This distinction becomes even more intriguing with the asthma-COPD overlap (ACO), which is described as a heterogeneous condition in which patients present clinical and inflammatory features of both asthma and COPD. The cardinal question is to what extent such distinctions have therapeutic implications. Despite using in smoking asthma the same medications as in non-smoking asthma, it is true that higher-dose ICS and dual bronchodilation are added to smoking cessation, which remains the critical intervention.

The concept of ACO has been one of the most ambiguous in the field of obstructive lung diseases, also reflected by the lack of a broadly accepted definition. Being a heterogeneous condition, ACO displays a challenge in terms of pathogenesis and risk factors. Patients with ACO experience a greater disease burden compared to patients with asthma or COPD alone, presenting accelerated decline in lung function, more frequent and severe exacerbations, higher risk of hospitalization, and poorer quality of life [14]. More studies are definitely needed in order to understand the natural course of ACO and to define biomarkers that could guide its diagnosis, prognosis and treatment.

A subset of asthmatic patients suffer from asthma with persistent airflow limitation that is not fully reversible (asthma with fixed airflow obstruction, FAO) and might not be smokers and/or do not suffer from COPD. Despite airway remodeling being recognized as a cause of FAO, the exact mechanisms underlying it are not yet fully elucidated [15]. To understand FAO, one might contemplate over the risk factors for it, such as early growth features, low initial FEV1, tobacco smoking, and blood and/or sputum eosinophilia. The need for a holistic approach including non-pharmacologic interventions such as smoking cessation, diet modification, and physical activity is essential in this group of asthmatics. It is interesting to note that the T2-high FAO phenotype responds equally satisfactorily to biologic treatments with mepolizumab, benralizumab, and omalizumab of asthmatic patients without FAO. [15].

Childhood asthma is a common heterogeneous chronic condition. In most cases, childhood asthma is associated with good asthma control. The use of currently approved add-on treatments with monoclonal antibodies for severe asthma in children has been shown to be effective in terms of asthma control and exacerbation rate. Omalizumab, mepolizumab, and dupilumab are licensed for children ≥ 6 years old, while benralizumab and tezepelumab for children ≥ 12 years old. [16]. There is lacking information regarding the long-term safety of biologics in the pediatric population, and more studies are needed. The questions regarding the optimal duration of biologic treatment in children with asthma and mainly the possibility of changing the natural course of the disease remain unanswered.

In the COVID-19 era, the prevalence of asthma among infected population varied significantly. Asthma patients are not at increased risk of SARS-CoV2 infection and not at increased risk of COVID-19-related death. This is actually more evident for allergic asthma, while non-allergic asthma presents a higher risk for severe COVID-19 outcomes [17]. Of note, underlying asthma severity is not a determinant of COVID-19 outcomes. This is supported by studies showing that severe asthmatics under biologic therapy did not demonstrate increased risk of severe COVID-19 disease and/or mortality. Accordingly, no modification in maintenance treatment including biologics is suggested for severe asthmatics during the COVID-19 pandemic.

It is more than evident that asthma is a heterogeneous disease, as stated in its definition, as it encompasses various phenotypes and endotypes with frequent overlapping. This phenotyping is essential and has been incorporated in the management of severe asthma. The clinician is triggered to reveal this phenotype with the evaluation of clinical features and biomarkers so as to address an effective and safe treatment that would lead to the diminution of exacerbations and increase control of the asthma. Exacerbations contribute significantly to asthma cost, and maintenance corticosteroids are associated with significant side effects. The paradigm of anti-IL-5 antibodies is indicative. A treatment that was initially judged as ineffective turned out to be an “asset” for many asthmatics with severe eosinophilic asthma. The key to success was two-fold: first, the proper choice of patients—those with persistent eosinophilic inflammation despite usual asthma therapy—and second, the targeting of the proper outcome, thus recuing exacerbations and dosage of oral corticosteroids.

Biologic therapies in severe asthma have proven to be life-changing and have paved the way for personalized treatment. A few questions remain: Do these treatments change the natural course of the disease? If this is so, may they be applicable in less severe forms of the disease? The newly risen concept of asthma remission gives hope for such a target. How long should we administer biologics? Can we combine them in overlapping phenotypes? Despite these questions, there is no doubt that advances in our understanding of the different immunologic mechanisms that drive airway inflammation have greatly improved the clinician’s ability to manage asthmatic patients in the clinic more efficiently by introducing biological treatments that target specific molecules in the inflammatory pathways. There is lack of evidence for targeted therapies in T2-low asthma, which remains a challenge. Despite that tezepelumab has shown some efficacy in T2-low asthma, there is an urgent need for biomarkers and new therapies for this asthma phenotype. Until then, it is obvious that the era of biologics has moved physicians toward a more personalized and precision-based approach and has shown the way to move in the future.
